# Treatment seeking behaviour in southern Chinese elders with chronic orofacial pain: a qualitative study

**DOI:** 10.1186/1472-6831-14-8

**Published:** 2014-01-25

**Authors:** Teresa SY Au, May CM Wong, Anne S McMillan, Susan Bridges, Colman McGrath

**Affiliations:** 1 Oral Rehabilitation, Faculty of Dentistry, University of Hong Kong, Prince Philip Dental Hospital, 34 Hospital Road, Hong Kong SAR, China; 2 Dental Public Health, Faculty of Dentistry, University of Hong Kong, Prince Philip Dental Hospital, 34 Hospital Road, Hong Kong SAR, China; 3 Centre for the Enhancement of Teaching and Learning/Faculty of Education, University of Hong Kong, Pokfulam, Hong Kong SAR, China

## Abstract

**Background:**

Chronic orofacial pain (OFP) is common in general adult populations worldwide. High levels of psychological distress and impaired coping abilities are common among Western people with chronic OFP but limited information was found in southern Chinese people. This study aimed to explore the perceptions and experiences of community dwelling elderly people with chronic OFP symptoms and their treatment seeking behaviour in Hong Kong.

**Methods:**

An exploratory qualitative interview study was conducted. Elderly people experiencing chronic OFP symptoms were invited to take part in an individual semi-structured interview. A total of 25 semi-structured interviews were performed for 25 participants.

**Results:**

Pertinent issues relating to the treatment seeking behaviour emerged from the interviews, many of which were inter-related and overlapping. They were organized into three major themes: (i) Impact of chronic OFP on daily life; (ii) Personal knowledge and feeling of chronic OFP; (iii) Management of chronic OFP. The participants were found to have the intention to seek professional treatment, but there were barriers which discouraged them continuing to seek professional treatment. They also received complementary treatment for chronic OFP, such as acupuncture, massage and “chi kung”. Moreover, a wide range self-management techniques were also mentioned. On the other hand, those who did not seek professional treatment for the chronic OFP claimed that they had accepted or adapted to the pain as part of their lives.

**Conclusions:**

This qualitative study observed that elderly people affected by chronic OFP symptoms in Hong Kong sought many different ways to manage their pain including traditional and complementary approaches. The role of the dentist in dealing with chronic OFP is unclear. Multiple barriers exist to accessing care for chronic OFP. The findings may be used to inform future chronic OFP management strategies in Hong Kong.

## Background

Orofacial pain (OFP) can be defined as pain related to the face and mouth regions and may involve both hard and soft tissues in these anatomical regions [1]. Chronic pain is a term used to describe pain that had persisted for 3 months or more, in accordance with the International Association for the Study of Pain definition [2]. The diagnosis and treatment of chronic OFP continues to be challenging even in contemporary modern dental practice. Anatomical structures in the head and neck region, mechanisms of referred pain, and underlying systemic and psychological pathology complicate diagnoses and management [3].

Chronic OFP is common in general adult populations worldwide with prevalence estimates ranging from 14-42% [4-8]. The adverse impact of OFP on sufferers’ lives can be considerable, especially if the pain is chronic [9-12]. Chronic OFP affects approximately 10% of adults and is more common in the elderly where 50% or more may be affected [13]. Experience of chronic OFP in the elderly has been found to vary between ethnic groups and appears to be more common in Asian elders [14,15]. The impact of chronic OFP also seems to vary between ethnic groups. However, the majority of people with OFP do not seek treatment [16]. There also appears to be an ethnic bias in treatment seeking behaviour with estimates of 40-46% in Western populations compared with around 20% in southern Chinese groups (including Hong Kong) [7,16-18].

High levels of psychological distress and impaired coping abilities are common among Western people with chronic OFP [6]. Whereas, in studies in southern Chinese people with chronic OFP of significant intensity, associated psychological distress was found to be limited and there were low levels of perceived need for treatment [19-21]. It has been proposed that southern Chinese people may have more effective coping strategies and more acceptance of pain than their Western counterparts [7,12,20].

The experience and consequences of chronic OFP have been explored predominantly using quantitative research methods [12-14,20]. Whilst the quantitative approach has yielded important information on the magnitude and impact of the chronic pain problem, it is not possible to investigate the perspectives, experiences and responses of individual patients.

The present study, therefore, aimed to explore the perceptions and experiences of southern Chinese community dwelling elderly people living in Hong Kong with chronic OFP symptoms and their treatment seeking behavior. Greater understanding of perceptions and experiences of chronic OFP within the local community has implications in defining ‘need’ in the local context, understanding the salience of chronic OFP to everyday life and functioning, and to provide a situation analysis of pathways to managing chronic OFP.

## Methods

By using the qualitative approach, the perceptions and experience of chronic OFP were collected by conducting individual semi-structured interviews with an interview schedule. In contrast to survey approaches, the semi-structured interviews enabled deeper insights regarding participants' knowledge and understanding the impact of OFP pain on their daily lives and their treatment seeking behaviors [22]. The study was approved by the Institutional Review Board of the University of Hong Kong/Hospital Authority Hong Kong West Cluster. Participants who took part in the study were provided with written informed consent.

### Participants

Hong Kong is often described as an ‘East meets West’ culture where knowledge, use and interest of traditional based approaches co-exist with conventional western-based medicine; particularly among older people [23]. This study population was community-based elderly people in Hong Kong who experienced chronic OFP. Inclusion criteria were: people aged 60 years and above with non-dental chronic OFP symptoms. Exclusion criteria were: communication difficulties; psychiatric disease including dementia and non-Cantonese speaking people.

Participants were a convenience sample of elderly people aged 60 years and above who were attendees at daytime social and community centres which provide leisure facilities and opportunities for socializing. In order to acquire a sample which was as sociodemographically as diverse as possible and with different characteristics of non-dental OFP of at least 3 months duration (i.e. chronic), subjects were recruited from different places within the Hong Kong Special Administrative Region (HKSAR), including Hong Kong Island and Kowloon. They were chosen from the publically available list of 57 social and community centres obtained from the website of the Social Welfare Department, the Government of the HKSAR (http://www.swd.gov.hk/doc/elderly/HP%20List%20of%20SE%20Oct_2013.pdf). The social and community centres were approached until enough eligible participants were recruited.

A short initial screening questionnaire was used to ask the potential participants regarding their experience of different types of non-dental chronic OFP symptoms, the duration and intensity of the symptoms and treatment seeking behaviour for chronic OFP. People who had, during the past month, experienced pain in their face, mouth or jaws which lasted for 1 day or longer and the pain had begun more than 3 months previously were eligible. The questions concerning chronic OFP symptoms comprised 9 items which did not include toothache (i.e. non-dental), viz. 1) pain in the jaw joint/s, 2) pain in the face just in front of the ear, 3) pain in and around the eyes, 4) pain in the jaw joint/s while opening the mouth wide, 5) sharp shooting pains across the face and cheeks, 6) pain in the jaw joints/s when chewing food, 7) pain in and around the temples, 8) tenderness of the muscles at the site of the face, and 9) a prolonged burning sensation in the tongue or other parts of the mouth.

### Construction of the interview schedule

The interview schedule was developed by including issues identified as potentially important from key literature in the field [9,15-17,20] and by conducting 2 focus groups.

The focus groups were designed to explore issues related to non-dental chronic OFP and treatment seeking for the pain. Elderly persons who had non-dental chronic OFP (with the use of the initial screening questionnaire mentioned above) were conveniently recruited from 2 social and community centres and one focus group session was held at each centre. They were invited to take part in the discussion of the focus groups. Each focus group contained 6 participants and the group discussions were conducted by a trained facilitator. Based on an interview guide, the facilitator encouraged participants to talk freely about their OFP perceptions and experiences, how and when professional care was sought for these conditions and how the conditions affected their daily living activities. Group discussions were conducted in Cantonese and each discussion lasted for around 1 hour. The conversations were audio-recorded and transcribed. Content analysis of the transcripts was performed by the facilitator to construct the interview schedule which consisted of the questions asked in the semi-structured interviews; no further analysis of the content was carried out. The constructed interview schedule included open-ended questions on the general experiences of chronic OFP, how the pain affected daily lives, pain characteristics (including severity, frequency and duration), knowledge and personal feeling of the pain and treatment seeking behaviour.

### Semi-structured interviews

A purposive sample of 25 elderly persons who had non-dental chronic OFP (with the use of the initial screening questionnaire mentioned above), were recruited from 18 social and community centres (excluding the 2 centres where the focus groups were conducted). Subjects with different age, gender, place of residence, educational attainment, oral health condition, self-rated oral health, pain characteristics and different treatment seeking behavior were invited to participate (Table 1).

**Table 1:** Characteristics of participants

**Demographic status**			**OFP characteristics**			
**Ref. no.**	**Age (Sex)**	**Education**	**Locations**	**Onset time**	**Duration**	**Severity**
[1]	78 (F)	Primary	- Right temple^a^	2 years	Seconds	Moderate
			- Right eye^a^	2 years		
			- Right face^a^	2 years		
[2]	65 (F)	Primary	- Tongue	10 years	Hours	Moderate
[3]	75 (F)	Primary	- Right temple	1 year	Minutes	Severe
			- Right jaw	1 year		
[4]	78 (F)	Secondary	- Tongue	5 years	Hours	Moderate
[5]	74 (F)	Primary	- Tongue	10 years	Minutes	Moderate
			- Right jaw	20 years		
			- Right chin	20 years		
[6]	60 (M)	Secondary	- Left face^b^	3 years	Seconds	Severe
			- Corners of Mouth^b^	3 years		
[7]	77 (F)	Primary	- Face^c^	10 years	Seconds to	Moderate
			- Eyes^c^	10 years	Hours	
[8]	71 (F)	Primary	- Jaws	3 years	Minutes	Severe
			- Tongue	3 years		
[9]	76 (F)	Primary	- Eyes	10 years	Minutes	Moderate
			- Jaws	10 years		
[10]	75 (F)	Primary	- Temples	6 months	Minutes	Mild
			- Palate	6 months		
[11]	73 (M)	Primary	- Right jaw	2 years	Minutes	Moderate
[12]	82 (F)	Primary	- Right temple^d^	2 years	Hours	Severe
			- Right face^d^	2 years		
[13]	80 (F)	Secondary	- Left eye^e^	15 years	Hours	Mild
			- Left face^e^	15 years		
			- Left chin^e^	15 years		
[14]	83 (F)	No	- Left eye	5 years	Minutes	Moderate
			- Left orbit	5 years		
[15]	68 (F)	Secondary	- Right temple^f^	12 years	Seconds	Severe
[16]	77 (M)	Primary	- Tongue	12 years	Minutes	Mild
[17]	80 (F)	Primary	- Tongue	4 months	Minutes	Mild
			- Left eye and left orbit	2 years		
			- Temples	2 years		
[18]	76 (F)	Primary	- Left eye and left orbit	1 year	Minutes	Moderate
			- Left temple	1 year		
[19]	83 (F)	Primary	- Left eye and left orbit	> 1 year	Seconds	Mild
[20]	76 (F)	Secondary	- Eyes	5 months	Minutes	Moderate
			- Nose	5 months		
[21]	80 (F)	Primary	- Temples	5 years	Hours	Moderate
[22]	82 (F)	Primary	- Right buccal cheek	20–30 years	Minutes	Mild
[23]	66 (M)	Tertiary	- Nose	4 years	Seconds to minutes	Moderate
			- Temples	4 years		
[24]	65 (F)	Tertiary	- Lichen planus	3 years	Seconds	Mild
			- Bilateral buccal cheek	3 years		
[25]	69 (F)	Primary	- Left face^g^	3 years	Minutes	Severe
			^-^ Left chin^g^	3 years		

Remarks: a = OFP was due to post-herpetic neuralgia; b = OFP occurred after brain tumour surgery; c = Neuropathic OFP; d = OFP was due to the recurrent trigeminal neuralgia (TN). The first episode of TN was in 20 years ago; e = OFP was due to TN; f = OFP was due to TN; g = OFP was due to TN.

The participants were interviewed individually and each discussion lasted for around 1 hour. The semi-structured interviews were conducted from October 2009 to January 2010. During each interview, body outline drawings were provided for the participants to mark pain locations in the orofacial region. Based on the interview schedule, a trained interviewer began the interviews by asking open-ended questions which were mainly on the general experiences of chronic OFP to allow the participants to raise any issues that they felt were important. Participants were encouraged to speak freely about how the pain affected their daily lives. Open-ended questions were asked on pain characteristics (including severity, frequency and duration), their knowledge and personal feeling of the pain and treatment seeking behaviour. Interviews took place until the dialogues become repetitive indicating that the exploration was saturated. The conversations were recorded for post-hoc transcription and analysis.

### Data analyses

The interviews were analysed using the Thematic Framework Approach that involved a multi-stage thematic analysis [24]. First, the audio-recorded interviews were transcribed verbatim in Chinese. The transcripts were reviewed by the trained interviewer line-by-line noting recurrent issues. Recurrent issues that emerged were then coded and indexed into different nodes, which contained related text in the same node so that any patterns and ideas could be easily identified. Another investigator verified the coding and indexing. The nodes were then organized into themes and sub-themes. Finally mapping and interpretation were achieved through discussion with the other investigators in the study. Quotations were selected to illustrate the observed patterns and interpretation. These selected quotations were then translated into English and reported in this paper.

## Results

### Sample characteristics

The characteristics of the participants are shown in Table 1, there were 21 female and 4 male participants with chronic OFP. Their age range was 65 to 83 years old. The extra-oral locations for chronic OFP were in the temple regions, eyes, face, jaw, chin or nose. The intra-oral locations for chronic OFP were in the tongue or buccal (cheek) mucosa. The participants had been suffering from chronic OFP for several years, some for more than ten years. In general, participants reported that their OFP was of mild to moderate severity.

### Themes

Numerous pertinent issues were obtained from the interviews, many of which were inter-related and overlapping. They were organized into three major themes: (i) Impact of chronic OFP on daily life; (ii) Personal knowledge and lay understanding of chronic OFP; (iii) Management of chronic OFP. A summary of the themes and sub-themes is shown in Table 2.

**Table 2:** Themes and sub-themes

**Theme**	**Sub-theme**
**1. Impact on daily life**	- Effect on normal daily activities
	- Effect on family and social lives
	- Effect on mood
**2. Personal knowledge and lay understanding of OFP**	
**3. Management of chronic OFP**	- Treatment obtained from physicians/ dentists/ traditional Chinese medical practitioner (TCM)
	- Use of complementary therapies
	- Use of self management techniques
	- Social support

#### Impact of chronic OFP on daily life

The participants expressed that their daily lives, including normal daily activities, social and family life and their mood had been adversely affected by the chronic OFP.

##### Effect on normal daily activities

Some participants reported that it was difficult to carry out daily activities and that their chronic OFP prevented them from doing previously routine things. For example:

“My eyes became very painful when I was looking at something for a long period of time. When my eyes felt painful, my vision would become blurred. For example, I could not read newspapers for a long period of time; otherwise my eyes would become tired and painful. So I could only read a few paragraphs from a newspaper.” (female participant, age 78, with moderate eye pain for 2 years due to post- herpetic neuralgia).

Another participant noted the change in her social behavior. As she became increasingly aware of her limited clarity in speaking, she deliberately reduced oral communication.

“A wound seems to be present on my tongue. My tongue would become very painful and could not move freely when I talk. This affects my speech because I could neither talk fluently, nor pronounce accurately…. So now I would rather not talk.” (female participant, age 65, with moderate tongue pain for 10 years).

Moreover, there appeared to be a relationship between the chronic OFP condition and the quality of sleep. Some participants noticed that they had poor sleep quality due to the pain. Those who had tongue pain and/or jaw pain reported that certain foods would trigger the pain, so they would eat very carefully and would select only certain types of food, one of them said:

“I get (tongue) pain when eating, I could not eat salty or hot food as it would make my pain worse… Now, I like to eat some less seasoned food, seldom eat deep fried food.” (female participant, age 78, with moderate tongue pain for 5 years).

##### Effect on family and social lives

The occurrence of chronic OFP had also impacted on the participants’ family and social lives. They mentioned that the chronic OFP had caused them to miss out on some family and social gatherings and/or activities or that it had affected their enjoyment of some events. They would reduce the public activities and would prefer to be alone when suffering from chronic OFP and not want to be bothered by anything or anyone. Some expressed concerns about how this affected their relationships with their family and friends. For example:

“I like playing mahjong very much, but when I get the pain (at the temple region), I could not concentrate and I would often lose the game……so I would not play mahjong when I felt unwell.” (female participant, age 68, with moderate pain at the temple region due to trigeminal neuralgia for 12 years).

“When I had the pain (temple region), I would not go to the community centre and I seldom tell other people about my pain, I would rather stay at home for some more rest.” (female participant, age 80, with mild pain at the temples region for 2 years).

An alternative, though less recurrent view was expressed by some participants that there was a need to get on with something, stressing the importance of distracting the chronic OFP and not letting the pain to govern them. They mentioned that they would not stay at home alone when the pain occurred, they would attended some social engagement or walk around outside the home. A participant emphasized going out of the house can relieve her pain:

“It is better to go out when the (eye and nose) pain occurs… I would feel less pain when I was being distracted. Moreover, in the daytime, my son goes out to work, I feel bored when I stay at home alone. It’s better to attend the community center in the daytime to spend my time.” (female participant, age 76, with moderate eyes and nose pain for 5 months).

##### Effect on mood

Participants routinely reported that the chronic OFP had badly affected their mood. Some mentioned that because their chronic OFP was unpredictable and considered to be untreatable by health professionals, they felt depressed, experienced self-regret, were worried, disturbed or anxious about the pain. They also expressed concern that they lacked of control over the course of the pain. One female participant even disclosed suicidal feelings because of her persistent chronic OFP:

“Sometimes I really want to die…Why do l live so long? I believe that the (jaw) pain could only be solved if I die. I always feel annoyed and depressed…Why is life so tough? I think it’s unfair for me to live so long and suffer from the pain!” (female participant, age 71, with both severe jaw and tongue pain for 3 years).

A contrasting view was expressed by those who reported that the chronic OFP would not affect their mood negatively. They claimed that their chronic OFP became part of their lives. Some claimed that they had accepted and/or adapted to the pain and could control it. This feeling seemed to be more common among those who had been suffering from chronic OFP for many years. Moreover, a participant said that it was better to forget the pain so that she could live happily as a healthy person:

“I have ignored the (eye, face and chin) pain. It’s better not to feel that you a “sick person”. I would prefer to live as a healthy person and do whatever I like, such as I would play, eat, go shopping and travel around as before.” (female participant, age 80, with eye, face and chin pain for 15 years due to trigeminal neuralgia).

#### Personal knowledge and Lay understanding of OFP

The participants who were worried about their chronic OFP conditions sought information from a variety of sources. They preferred to obtain advice directly from their physicians; however, their physicians often did not make a detailed examination, or give a formal diagnosis or an explanation for the chronic OFP. On the other hand, traditional Chinese medical practitioners generally diagnosed the chronic OFP as the cause of “internal heat” inside the human body.

Participants generally reported that they obtained more information about their chronic OFP from reading some informative books, magazines, through the internet or advice from their friends and family. Therefore, they had developed their own ideas about different aspects of their chronic OFP, including diagnosis and underlying causes. Some of them even worried about the underlying causes of the pain being something very serious, such as tumors, stroke or glaucoma. They also identified that, in some cases, triggers for their chronic OFP might be as infection, carious teeth, stress, diet and lifestyle factors. For example:

“I think that “internal heat” will affect the nervous system. After I knew I had the neuropathic pain from my physician, I avoided eating spicy food, such as pepper because I believe that the spicy food would create the “internal heat” which would stimulate the neuropathic pain.” (female participant, age 77, with moderate neuropathic pain around the face and eyes for 10 years).

“I do not have any knowledge about the facial pain, I think that the (nose and eyes) pain was caused by the toothache. Because my eyes started to have pain after I have had toothache… so I believe that all my facial pain was radiating from the carious tooth.” (female participant, age 76, with moderate eyes and nose pain for 5 months).

#### Management of chronic OFP

##### Treatments obtained from physicians/dentists/traditional Chinese medical practitioners (TCM)

The participants would generally seek help from health professionals as the first strategy for treatment of their chronic OFP. However, they felt there was a lack of special clinics for the treatment of OFP in Hong Kong. The participants usually sought professional help from physicians, traditional Chinese medicine and/or dentists. However, they seldom sought treatment from physicians just solely for the chronic OFP. They would prefer to consult physicians about their chronic OFP at the time when they had follow-up and/or consultation for other systemic/general problems. In these situations, they had some bad experiences reporting that the physicians were disinterested or dismissive of their problems. This discouraged them from continuing to seek treatment from the physicians. One of them said:

“Last time when I had a regular check-up of blood pressure at the government clinic, I also asked the physician about the reason and treatment for my jaw pain, but he was unwilling to treat my problem and ignored my question about my jaw pain.” (female participant, age 74, with moderate jaw pain for 20 years).

Some of them reported that they had approached many different health professionals (including physicians, TCM and/or dentists) directly about their chronic OFP; however, they did not find any solutions for their pain. The physicians most often only gave analgesics to relieve the pain, instead of asking them any detailed history or providing a detailed examinations and diagnosis. Although they reported that the analgesics could help them to relieve some of the pain, they expressed some concerns about the drawbacks of the medications. They were especially concerned about the side effects of the analgesics, such as gastric problems and/or dizziness. People who were worried about the side effects of the analgesics claimed that they would only take the analgesics if the OFP became very severe and they could not tolerate it. For those who had been taking analgesics for their chronic OFP for a long time, they found that those medications were not as effective as they had been initially. Moreover, drug interactions appeared to be a problem for those participants who were also taking a number of other medications for their systemic disease. Some of them said:

“I visited the physician for my jaw pain. He prescribed me some analgesics. I (would) seldom take the medications. As I often have stomach ache, I am afraid that the medication will affect my stomach.” (male participant, age 73, with moderate jaw pain for 2 years).

“I would not take the analgesics because these drugs could not treat my OFP. My physician prescribed me some analgesics, he told me to take them if the pain was severe. But I believe that the analgesics have more cons than pros, so I would rather bear the pain instead of taking them.” (female participant, age 77, with moderate neuropathic pain in the face and eyes for 10 years).

In contrast, those who occasionally took analgesics to relieve their chronic OFP claimed that they depended very much on those analgesics. For example, A 66 year old participant mentioned that he did not worry about the side effects of the analgesics because the physicians told him that they were negligible.

Those who consulted Traditional Chinese Medical practitioners (TCM) reported that they were told that their chronic OFP was due to the “internal heat” inside their body and they were prescribed some Chinese medicine to release the “internal heat”. However, they claimed that the internal Chinese herbal medicines were not very effective for relieving the chronic OFP. One of them said that she could not take the Chinese medicine because of its side effects:

“I am suffering from rheumatic heart disease (RHD) and have taken medications for a period of time. I could not take the Chinese medicine because last time I got nose bleeding after taken it. Then my physician told me that it was due to the drugs interactions between the Chinese medicine and the medications for the RHD.” (female participant, age 78, with moderate tongue pain for 5 years).

Participants were less likely to seek treatment from dentists for their chronic OFP. Some thought that the chronic OFP was not related to dentistry, so they had not thought of seeking treatment from the dentists. Moreover, some claimed that the dental treatment fee was very expensive and they could not afford it. For example:

“I have consulted several physicians and traditional Chinese medical practitioners (for my tongue pain). However, they couldn’t solve the problem… the Chinese herbs did not help and the physicians could only give me some vitamins. Now I was dispirited about continuing seeking treatment for the pain. On the other hand, I have not consulted dentists about my tongue pain because I thought that it was not a dental problem. I don’t know whom should I seek treatment from.” (female participant, age 65, with moderate tongue pain for 10 years).

“I consulted a physician for my jaw pain before, he suggested that I seek treatment from a dentist. However, I cannot afford the dental treatment fee because it is very expensive. I hope that the government can provide free dental treatment service for me.” (female participant, age 71, with severe jaw pain for 3 years).

The participants who never sought any treatment from the health professionals claimed that their OFP was not a problem for them and the pain did not have a great impact on their daily lives. They could function normally in daily duties and the pain did not affect their social life and mood. The chronic OFP they suffered was mild and they claimed that they could control it. For example:

“The (left eye) pain started last year, I haven’t sought any treatment for the pain. Because it disappears if I close my eyes for a while and I avoid from looking at an object for a long time.” (female participant, age 83, with mild left eye and orbit pain for more than 1 year).

##### Use of complementary therapies

Some participants had tried complementary therapy after they found that the health professionals were not so effective in solving their chronic OFP. The complementary therapies were often recommended by their friends or relatives. Acupuncture and massage were the most frequently mentioned therapies. However, some commented that these therapies were costly, while others had stopped using them because they found that they were not so effective or just for short term pain reduction. As one of them said:

“I had tried acupuncture many times before… The (tongue and jaw) pain was relieved after the first few times but I found that it was not effective afterwards. Acupuncture was just effective for short term pain relief.” (female participant, age 74, with moderate tongue pain for 10 years and jaw pain for 20 years respectively).

“Later, I had massage therapy; however, I found that it was not very effective to relieve the (face and chin) pain…Afterwards, my daughter recommended a famous traditional Chinese medical practitioner to me for the acupuncture treatment. After I got the twelve courses of acupuncture treatment, I found it was also not effective.” (female participant, age 69, with face and chain pain due to trigeminal neuralgia for 3 years).

A contrasting view was expressed by a participant who had severe pain in the jaw and temple region. She reported that “chi kung” was very effective for treating her pain. Before she sought “chi kung” treatment, she had consulted multiple health professionals, but they were not very helpful. Then her friend suggested that she seek treatment from a master of “chi kung”. She said:

“I remember I had treatment of my (jaw and temple region) pain from the master of “chi kung” for two years. I visited him everyday at the beginning, but now I visit him once per week only for regular follow-up. Although most people do not believe in “chi kung”, I believe in it because it really could help me to relieve my (jaw and temple region) pain.” (female participant, age 75, with severe pain at the jaw and temple region for 1 year).

##### Self management techniques

Apart from treatment from the health professionals and having complementary therapies, participants described a number of other techniques which they found helpful for alleviating their OFP. Again, some of these methods were suggested from their friends or relatives. Common strategies included application of Chinese herbal oil, self massage, cold or warm compression, using medical pads, nutrition, taking more rest, physical exercise and over-the-counter medications. Here are some of the conversations from the participants:

“When it (jaw and face) was painful, I would use my hands to press onto the painful regions to relieve the pain… Everyday, I also do massage on my face in the mornings and at nights.” (female participant, age 75, with severe pain at the jaw and face for 1 year).

“When the (jaw) pain was very severe; I would occasionally apply herbal oil onto the painful region and it was quite effective to relieve the (jaw) pain.” (female participant, age 71, with severe pain at the jaw for 3 years).

“Everyday I use medical pads to stick onto the painful regions (right side temple and face). I think it is very useful for relieving the pain. Moreover, when I sleep, I put a towel under the right side of my head to prevent the pillow from touching the painful regions.” (female participant, age 82, with severe pain at the face and temple region for years due to recurrent trigeminal neuralgia).

On the other hand, some described less conventional techniques, such as doing some other activities to distract their focus on the chronic OFP, such as playing mahjong or number cards, swimming, singing or doing meditation. One of them said:

“I think the most wonderful time in each day is when I am in the swimming pool. …. I like to swim slowly inside the pool. At the time when I am floating in the water, I do not feel any (temple and nose) pain.” (male participant, age 66, with moderate pain at the nose and temple region for years).

##### Social support

The participants did not want to mention their chronic OFP to their family and friends because they did not want them to worry and/or they felt others would not understand their pain and could not help them. For example:

“I have never talked about my (right jaw) pain to the others because I think that they cannot help me to solve the problem. Even for my wife, I have never told about my pain as I don’t want her to worry about me.” (male participant, age 73, with moderate pain at the jaw for 2 years).

In contrast, other participants would share their concerns about their pain with others. In some cases, friends helped them to relieve the pain. For example, one of the participants who had trigeminal neuralgia for 3 years mentioned that she had consulted many health professionals to treat the pain in the chin and face regions, but they were not helpful and she became very desperate. However, her friend recommended a Chinese medicine which she found to be was very effective for relieving the pain. She said:

“My friend heard that I had OFP, she gave me some Chinese herbal tea and claimed that it could relieve the “internal heat” inside my body. After I drank the tea, the pain (face and chin) seemed to be reduced. I took it every morning for one month. To my surprise, the pain (face and chin) would be seldom occurred now.” (female participant, age 69, with severe pain at the face and chin for 3 years due to trigeminal neuralgia).

On the other hand, another participant had some bad experiences from the “help” of her friend. She said:

“My doctor was unwilling to prescribe the medication for the pain at my right eye. One of my friends who had also suffered from Herpes zoster infection in the past suggested I buy a topical medication. Therefore, one day I bought this topical medication and apply around my eye region in the morning. However, in that afternoon, I found that my eye became very red… I immediately visited my doctor to seek treatment.” (female participant, age 78, with moderate eye pain for 2 years due to post-herpetic infection).

**Figure 1:**
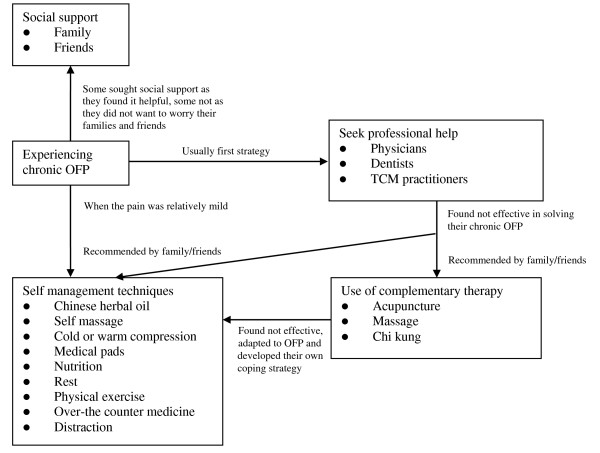
Experience, adaptation and management of chronic OFP.

Figure 1 summarizes the experience, adaptation and management of chronic OFP. When the elders reported experiencing chronic OFP, their first strategy was usually to seek professional help from physicians, dentists and/or TCM practitioners. Complementary therapy and/or self-management techniques recommended by family/friends were adopted when they viewed the professional help to be ineffective in solving their chronic OFP. However, when this pain was relatively mild, they undertook self management techniques to cope with their chronic OFP. Social support was sought by some elders as they found it helpful but not for the others as they did not want to worry their families and friends.

## Discussion

This qualitative, interview-based research study involved southern Chinese elders from the general population in Hong Kong who were suffering from different types of non-dental chronic OFP. The purpose of the qualitative approach is to contribute the conceptual and theoretical knowledge on particular issues can be learned from individual life experiences and perceptions [22].

According to previous quantitative research, OFP symptoms were found to have a significant detrimental effect on functional, psychosocial well-being, daily life activities and lowered the quality of life of the Chinese elders [15,20,25,26]. However the majority of southern Chinese elderly people did not seek professional treatment for chronic OFP: only 27% with OFP symptoms sought professional treatment [15,27]. The likelihood for treatment seeking for OFP increased with the number of days when OFP was experienced [27]. However, in this study, we found that the participants who were suffering from chronic OFP were keen to consult health professionals in the hope of relieving the pain symptom. On the other hand, there were only a few of them who had never sought any professional treatment for the chronic OFP. The most likely reason for not seeking treatment was that the pain was relatively mild in nature and it did not have a great impact on their daily lives. They claimed that they could control the pain by their own coping strategies and they had already accepted/ adapted to the pain as part of their lives.

People with chronic OFP often have sought help from multiple health professionals for symptomatic pain relief. Because there is a lack of a “specialized chronic OFP clinic” in Hong Kong, we found that some of the participants did not know where and from whom they should seek treatment. Among the available choice of health professionals, most of them preferred to seek treatment from a physician rather than a dentist. This finding was in agreement with our previous finding [15,27] and another study in the United Kingdom [28]. The concepts of the clinical role of physician and dentist were usually determined by the patients’ experiences and perceptions as well as the influence from their family and peers [29]. They regarded physicians as being better trained to diagnose and treat symptoms that are of non-dental origin [28]. It is relevant that over half of the dental graduates from the University of Hong Kong felt that they were less well equipped to relieve chronic OFP [30]. This might indicate that there was a lack of confidence of the dentists in the diagnosis and management for chronic OFP. This situation should be improved via undergraduate and postgraduate dental training and continuing development professional courses, as well as improved patient awareness of chronic OFP [28,30].

According to a survey in Hong Kong, some barriers exist to accessing the oral health care services. The problem related to the cost of oral health care services could be due to many reasons such as the price information not being available, dental services not being affordable, or a low level of appreciation or value on the cost of care [31]. From our interviews, it was clear that some participants were worried about issues like pain and discomfort during the dental treatment and some were concerned about the cost of dental treatment.

When consulting physicians regarding their OFP, it was unexpected to find that our participants most often did this indirectly. They preferred to consult the physicians during follow-up and/or consultations for other systemic problems, especially at government medical clinics. However, in these situations, the participants complained that the physicians were uninterested and not willing to treat the chronic OFP problems. In Hong Kong, the dissatisfaction on the short consultation but long waiting times, no stable doctor and no freedom to choose physicians to facilitate continuity of care in seeking health care services in the public sector have been already reported [32]. These factors are likely to discourage people from seeking treatment for chronic OFP.

When a curative treatment is not available for chronic OFP, people often expect to be given analgesic medications (“pain killers”) for pain relief. Although analgesics could effectively relief acute pain in the short run, their efficacy in treating chronic pain is probably marginal and controversial [33]. In our study, the majority of participants were reluctant to take the prescribed analgesic because they were concerned about the side effects. Some mentioned that the analgesic could only relieve the pain symptoms temporarily, but it could not cure the OFP completely. Moreover, some participants who had other systemic diseases were concerned about the possibilities of drug interactions between the analgesics and their existing medications. However, considering the widespread use of analgesics, the overall incidence of serious drug-drug interactions involving the analgesics has been relatively low [34]. Thus, even with different available biomedical treatments for chronic pain, more effective complementary and alternative treatments are needed.

Some participants consulted TCM practitioners for the treatment of chronic OFP. They received either internal Chinese herbal medication and/or complementary treatment. TCM has been known for more than 5,000 years and the belief in Chinese medicine is still ingrained in the general Hong Kong population. According to a previous survey, ten percent of the people in Hong Kong would consult TCM practitioners for their illnesses [35]. However, TCM is considered to be a complementary and alternative medicine (CAM) in many Western countries [36].

“Chi kung” and “Tai Chi” are closely associated with TCM but typically considered as complementary treatments. One of the participants who had received “chi kung” treatment claimed that it was a very effective in relieving her chronic OFP. “Chi kung” or “Qigong” is important in the cultural heritage of China and describes various Chinese systems of ways to improve health both physically and mentally [37]. “Chi kung” has also been found previously to relief chronic OFP [38].

On the other hand, participants who had received acupuncture and massage claimed that those treatments were not as effective in to relieving their pain. Some reported that acupuncture treatment was effective in short-term pain reduction only. Acupuncture has also been shown to provide a significant short-term pain relief in patients with chronic OFP [39]. However, there was no evidence to support massage therapy was effective to relieve chronic OFP [40].

Apart from seeking treatment from different health professionals, participants also developed their own coping strategies and described a wide range of various self management techniques which were quite effective in the relief of chronic OFP in most situations. Some of the techniques were suggested by their friends or family. The most commonly used technique was application of the Chinese herbal oil onto the painful region. The herbal remedies employed natural plant preparations for therapeutic effects [40]. Uses of herbal remedies to reduce facial pain have been described, but there is generally insufficient evidence to support its use for chronic pain relief [41].

The qualitative study approach provided a deeper contextual understanding of the experiences and practices of older adults affected by chronic OFP. Another strength was the location of the study as the accessing of social and community centres increased diversity thus enabling wider insights into the multitude of care pathways (both conventional and traditional) when compared to clinic-based studies. Additionally, inclusion of a community sample as opposed to a clinical sample overcame possible biases of perceptions among a treatment-seeking study group. Data saturation was reached after the 25 interviews (around 1 hour each) and so the sample size was deemed adequate. In qualitative studies, the focus is on context and meaning rather than building a representative view of the population and this approach has limitations. A further limitation is that no clinical objective assessment of chronic OFP was undertaken to confirm chronic OFP diagnoses.

These findings have implications in providing greater ‘in depth’ understanding of the elders experience of chronic OFP with implications to inform the need for services including multidisciplinary specialty clinics. The study highlights the issues faced by elders affected by chronic OFP in the local context (and potentially other populations) and the need to address this problem through community means.

## Conclusions

In conclusion, this qualitative study observed that people with chronic OFP symptoms in Hong Kong seek many different ways to manage their pain including traditional Chinese and complementary approaches. The role of the dentist in the management of chronic OFP appears unclear. A number of barriers exist to accessing care for OFP. The present findings may be useful to inform future chronic OFP management strategies in Hong Kong.

## Competing interests

The authors declare that they have no competing interests.

## Authors’ contributions

TSYA collected the research data and conducted all the analyses under the supervision of ASM and MCMW. SB provided specialist advice on qualitative methodology. CM provided expert advice on oral health-related quality of life issues. All authors contributed to the preparation of the manuscript. All authors have read and approved the final version of the manuscript.

## Pre-publication history

The pre-publication history for this paper can be accessed here:

http://www.biomedcentral.com/1472-6831/14/8/prepub
